# Tests of the chromatographic theory of olfaction with highly soluble odors: a combined electro-olfactogram and computational fluid dynamics study in the mouse

**DOI:** 10.1242/bio.047217

**Published:** 2019-10-24

**Authors:** David M. Coppola, Emily Fitzwater, Alex D. Rygg, Brent A. Craven

**Affiliations:** 1 Department of Biology, Randolph-Macon College, Ashland, VA 23005, USA; 2 Department of Ecology and Evolutionary Biology, UCLA, Los Angeles, CA 90095, USA; 3 Department of Mechanical and Nuclear Engineering, The Pennsylvania State University, University Park, PA 16802, USA

**Keywords:** Odor discrimination, Sorption, Olfactory receptors, Maps

## Abstract

The idea that the vertebrate nasal cavity operates like a gas chromatograph to separate and discriminate odors, referred to herein as the ‘chromatographic theory’ (CT), has a long and interesting history. Though the last decade has seen renewed interest in the notion, its validity remains in question. Here we examine a necessary condition of the theory: a correlation between nasal odor deposition patterns based on mucus solubility and the distribution of olfactory sensory neuron odotypes. Our recent work in the mouse failed to find such a relationship even across large sorption gradients within the olfactory epithelium (OE). However, these studies did not test extremely soluble odorants or low odor concentrations, factors that could explain our inability to find supporting evidence for the CT. The current study combined computational fluid dynamics (CFD) simulations of odor sorption patterns and electro-olfactogram (EOG) measurements of olfactory sensory neuron responses. The odorants tested were at the extremes of mucus solubility and at a range of concentrations. Results showed no relationship between local odor sorption patterns and EOG response maps. Together, results again failed to support a necessary condition of the CT casting further doubt on the viability of this classical odor coding mechanism.

## Abstract (precis)

**Summary:** This paper casts doubt on the classical chromatographic theory of olfaction, showing there is no correlation between olfactory receptor spatial layout and odor solubility patterns, a necessary condition of the theory.

## INTRODUCTION

More than a half century ago, Maxwell Mozell (1964, 1966, 1970) made a series of observations in the frog that prompted him to liken the workings of the vertebrate nasal mucosa to a gas chromatograph, an analogy that crystalized ideas about a mechanism of odor discrimination based on receptor spatial layout suggested earlier by his mentor, the Nobel laureate, Lord Adrian (1942, 1950, 1954). For Adrian, his proposed mechanism rendered olfaction consistent with the other exteroceptive sensory epithelia like the retina, cochlea and somatosensory components of the skin, in having a spatial logic. The concept in its simplest form holds that upstream portions of the nasal mucosa selectively capture highly mucus-soluble odors, allowing relatively less mucus-soluble odors to pass farther along the airflow path. The directing of odors in this way to specific parts of the olfactory epithelium (OE) is thought to aid in odor discrimination, though the details of this part of the theory have never been adequately elaborated (reviewed by Schoenfeld and Cleland, 2006). However, it is clear that the chromatographic theory (CT) predicts a relationship between odor sorption patterns, which are dictated by odor mucus solubility and nasal airflow, and the distribution of olfactory sensory neuron receptive-field types.

In the intervening decades, an expansive body of empirical work has accrued supporting the CT (e.g. Mozell, 1970; Kent et al., 1996; Scott et al., 1996, 2000, 2014; Scott and Brierley, 1999; Rojas-Líbano and Kay, 2012; reviewed by Schoenfeld and Cleland, 2005, 2006). By contrast, our recent studies in the mouse, which compared computational fluid dynamics (CFD) simulations of odor sorption patterns with electro-olfactogram (EOG) measurements of olfactory receptor response maps, largely failed to support the most critical predictions of the CT (Coppola et al., 2017). For example, while we found that most water-soluble odors evoked stronger responses at a dorsal-central recording site on the OE than in the periphery, local sorption gradients along individual turbinates were uncorrelated with OE response gradients. Notably, this held true even in areas of the OE where there were marked sorption gradients for a particular odor. However, these studies typically employed only one or, at most, two stimulus concentrations and tested a limited set of highly soluble odors, the latter a chemical class about which the CT has its clearest predictions.

Here we report results that further test the CT's validity by mapping mouse receptor responses using the EOG and compare these response maps to CFD predictions of sorption patterns in the nasal cavity. The stimulus set used for EOG recordings, unlike previous studies, included a dilution series and odors at the extreme of mucus solubility. The use of a dilution series allowed us to test the possibility that high sensitivity receptors play an outsized role in odor detection i.e. that we missed EOG/CFD correlations in our previous studies because we used high concentrations, which recruit non-specific receptors. The use of extremely mucus-soluble odors allowed us to test the possibility that the CT operates only for highly mucus-soluble odors, a reasonable restriction given large sorption gradients of this class of stimuli.

Recordings targeted the OE covering the dorsal branch of endoturbinate II, where CFD simulations reveal marked gradients in the sorption of mucus-soluble odors. We also immunolabeled the dorsal–central receptor zone, often called Zone 1, which selectively expresses NADPH quinone oxidoreductase (NQO1) so we could compare its boundaries to response maps measured with the EOG (Gussing and Bohm, 2004; Kobayakawa et al., 2007**)**.

Results confirm our previous observations that, contrary to the CT, OE response gradients do not correlate with local odor sorption patterns, no matter the concentration or estimated mucus solubility of the stimulus. Importantly, CFD simulations also reveal that above some level of mucus solubility, odor sorption patterns change very little among odors. Thus, our sorption simulation results should apply to any highly mucus-soluble odor. Further, we show that there is a sensitive region on the OE for fatty acids that corresponds to the dorsal–central area known to contain class I olfactory receptors (Niimura and Nei, 2007; Niimura, 2014). Together, our results are incompatible with a foundational premise of the CT that there exists an ‘inherent’ pattern of sensory neuron receptive-field types configured to take advantage of local odor sorption patterns (Moulton, 1976; Youngentob et al.; 1995; Kent et al., 1996; Schoenfeld and Cleland, 2006). Our demonstration of a sensitive region for fatty-acids odors on the OE is consonant with a growing body of evidence that the dorsal–central zone is specialized for different functions than the ventral (peripheral) zone, but it is not specialized for water-soluble odors, per se (Kobayakawa et al., 2007).

## RESULTS

### NQO1 immunolabeling

**Fig. 1.**
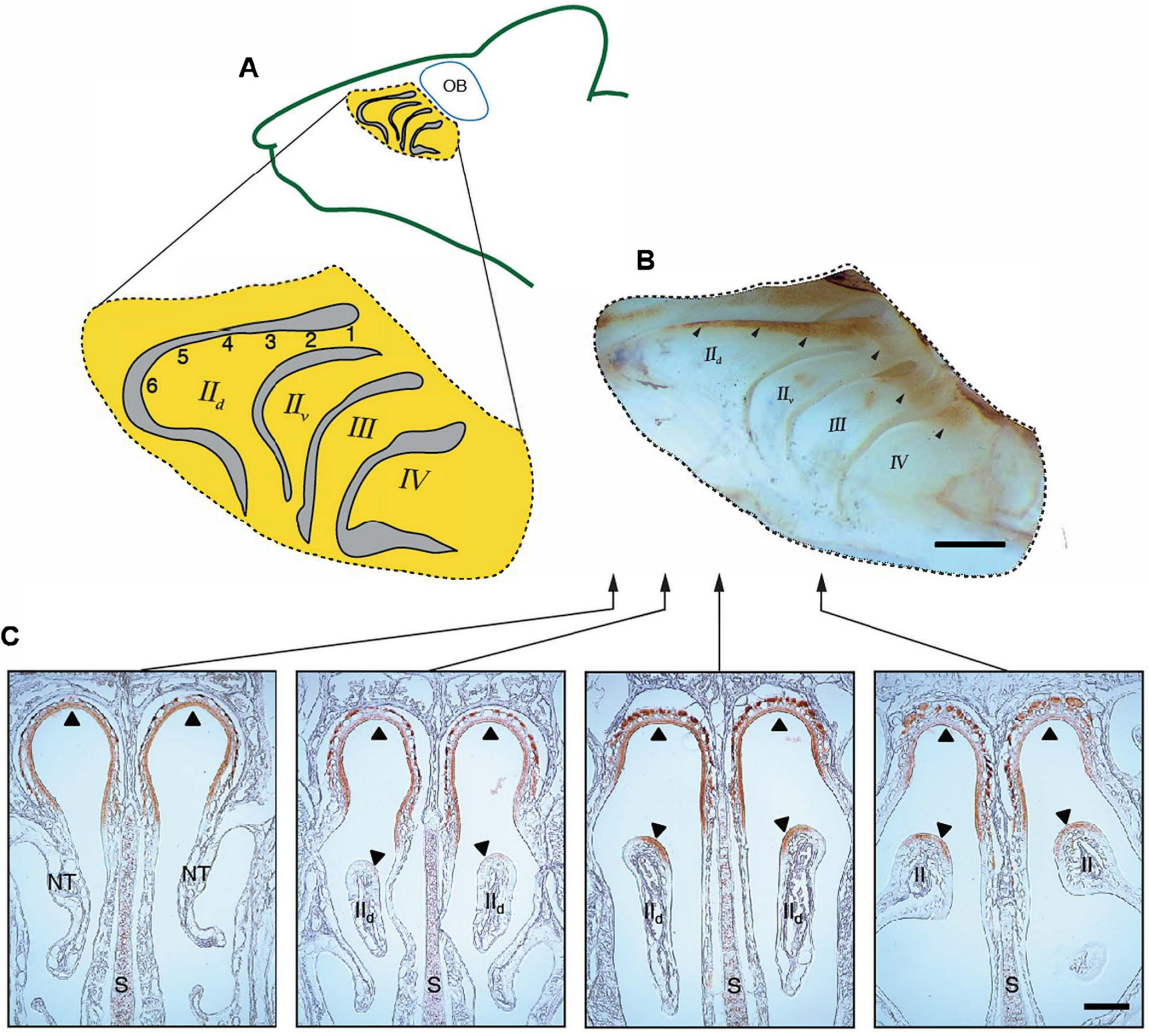
**Recording locations and receptor zone boundaries.** (A) Midsagittal drawing of endoturbinates (rostral to the left). The primary EOG recording locations on the dorsal branch of endoturbinate two (II_d_) are designated with numerically. Roman numerals designate endoturbinates using standard nomenclature (Coppola et al., 2017). (B) Micrograph of midsagittal view of whole mount showing endoturbinates with NQO1 immunolabeling revealing boundaries of dorsal-central (Zone 1; *n*=6 mice). Note brown label margins (arrowheads) and compare to recording locations in A. (C) Immunolabeling for NQO1 of coronal sections through a portion of the nasal cavity (*n*=5 mice). Arrowheads show limited distribution of label in dorsal meatus (top) and dorsal portion of turbinate II_d_. Arrows show approximate rostrocaudal location of sections using the whole mount as a reference. S, nasal septum; NT, nasal turbinates; endoturbinates are labeled with Roman numerals by convention (Coppola et al., 2017). Scale bars: 1 mm (top), 0.5 mm (bottom).

Our NQO1 immunolabeling, consistent with previous reports, revealed the dorsal–central olfactory epithelial zone (Zone 1) as occurring along the dorsal crown of the caudal ∼75% of endoturbinate two (II_d_) with the zone extending ventrally near this turbinate's caudal terminus (Fig. 1B,C; Ressler et al., 1993; Sullivan et al., 1996; Gussing and Bohm, 2004; Iwema et al., 2004). Thus, our recording locations one to four are in the dorsal zone. Location five appears to be on or near the borderline of the dorsal and ventral zones while location six is in the ventral zone. Note from the labeled midsagittal view of a nasal cavity whole mount (Fig. 1B) that the dorsal zone is limited to the extreme caudal areas of the other endoturbinates (II_v_, III and IV). Our sampling of six whole-mount and five cryostat-sectioned mice suggest that the NQO1 pattern, and thus by inference the boundaries of the dorsal olfactory receptor zone, is quite similar across animals.

### Comparison of simulated odorant sorption patterns and EOG response maps

**Fig. 2.**
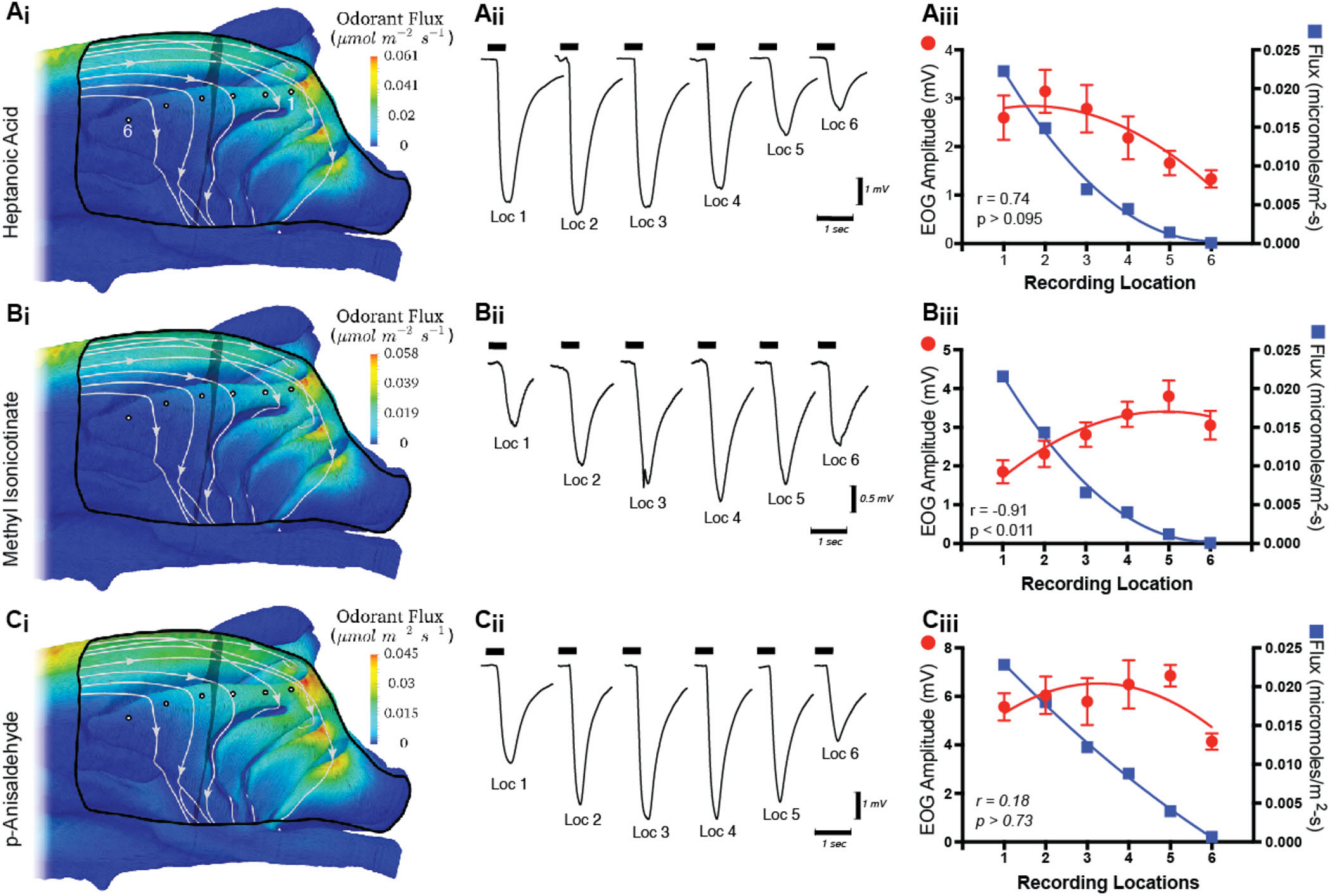
**CFD simulations and EOG results.** (A_i_,B_i_,C_i_) CFD simulations of airflow paths and odor deposition (flux) patterns are illustrated in the olfactory recess for three odors chosen because they have contrasting EOG response profiles (see Results). Flow patterns are illustrated with streamlines calculated from the CFD solution. In these medial views note that a region (black line) of the septum has been digitally removed to reveal the underlying endoturbinates. The EOG recording locations on endoturbinate II_d_ are shown as black-outlined white circles. Location one is caudal-most (right) and location six is rostral-most (left). (A_ii_,B_ii_,C_ii_) Sample raw EOG traces from individual animals at each of the standard recording locations in response to 0.1% concentration of stimulus. The thick horizontal segments above the traces show when the stimulus was turned on. (A_iii_,B_iii_,C_iii_) Mean (±s.e.m.; *n*=>9 mice per odor) EOG amplitudes at different recording locations are shown with red lines and symbols for three odorants [see odors and recording locations in A_i_,B_i_,C_i_. For comparison to the EOG responses, odorant flux values were extracted from the CFD simulations of odorant deposition at the recording locations and are plotted in blue (right vertical axes)]. Note that ordinates have different scales to account for the different odor intensities so as to highlight EOG and CFD relationships. Pearson-r correlations and *P*-values are shown for each graph.

Surface contours of odorant flux in the olfactory recess from CFD simulations are shown for three of the nine odorants used in this study (Fig. 2). Contours for a fourth mucus-soluble odor, acetophenone, are available from our previous study (Coppola et al., 2017). Rygg and colleagues (2017) have recently shown in canines that CFD-simulated sorption patterns within the nasal cavity are largely similar for odorants with mucus solubility greater than that of acetophenone. Our simulations confirm this observation for mice given that the sorption patterns for acetophenone (Coppola et al., 2017), p-anisaldehyde, methyl isonicotinate and hepantoic acid, (Fig. 2), chemicals with a 400-fold range of mucus solubilities, have virtually indistinguishable sorption patterns. The pattern, which would seem to be characteristic for all highly soluble odorants, shows scant odor deposited along the rostral one-third of endoturbinate II_d_. This is because the air that flows across this portion of the turbinate previously passed along the dorsal surface of the maxilloturbinate before flowing through the ventral aspect of the dorsal meatus and into the olfactory recess (Coppola et al., 2017). As a result, the odor concentration in the air that flows over the rostral one-third of endoturbinate II_d_ is nearly zero for highly soluble odors due to upstream respiratory filtering. In contrast, air that flows over the caudal portion of endoturbinate II_d_ has a much higher odor concentration, as this air stream previously flowed exclusively through the dorsal meatus, where there is less surface area and residence time for odorant deposition, the latter owing to the higher flow speeds in this straight conduit that bypasses the convoluted respiratory region. Because of this higher odorant concentration, the caudal portion of endoturbinate II_d_ has comparatively large values of odorant flux that increase substantially toward the caudal-most part of the turbinate. Quantitative values of odorant flux for p-anisaldehyde, methyl isonicotinate and heptanoic acid are shown in Fig. 2A_iii_, B_iii_ and C_iii_, where values are plotted from the CFD simulations interrogated at each of the EOG recording sites along endoturbinate II_d_. Note the steep drop-off in flux for each odorant as one considers caudal to rostral recording locations (one to six).

In contrast to the similar odor sorption patterns across test odors, the EOG response gradients recorded from the same locations were remarkably variable from odor to odor. For example, heptanoic acid showed a significant negative trend moving from caudal to rostral recording locations (W=−49, *n*=10, *P*<0.01) that was positively but non-significantly correlated with the flux gradient (r=0.74; *P*>0.09; Fig. 2A_iii_). Methyl isonicotinate, an odor that is more mucus soluble than heptanoic acid (Table 1), had a positive trend (W=36, *n*=8, *P*<0.01) in average EOG responses moving from caudal to rostral recording locations resulting in a significant inverse correlation with odorant flux (r=−0.91; *P*<0.01; Fig. 2B_iii_). In a third example, p-anisaldehyde, with mucus solubility nearly three log units less than methyl isonicotinate, had a flat profile (W=−17, *n*=9, *P*>0.35) of average EOG responses moving from caudal to rostral recording locations that was uncorrelated with the flux gradient for this odor (r=−0.18; *P*>0.7; Fig. 2C_iii_). The failure to observe any consistent relationship between local odor flux patterns and EOG gradients for highly soluble odorants is consistent with our previous studies, which included odorants with a wide range of mucus solubilities (Coppola et al., 2017).

**Table 1.**
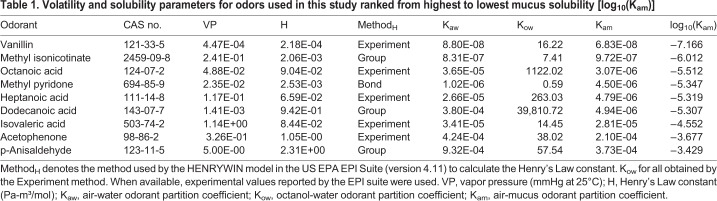
**Volatility and solubility parameters for odors used in this study ranked from highest to lowest mucus solubility [log_10_(K_am_)]**

**Fig. 3.**
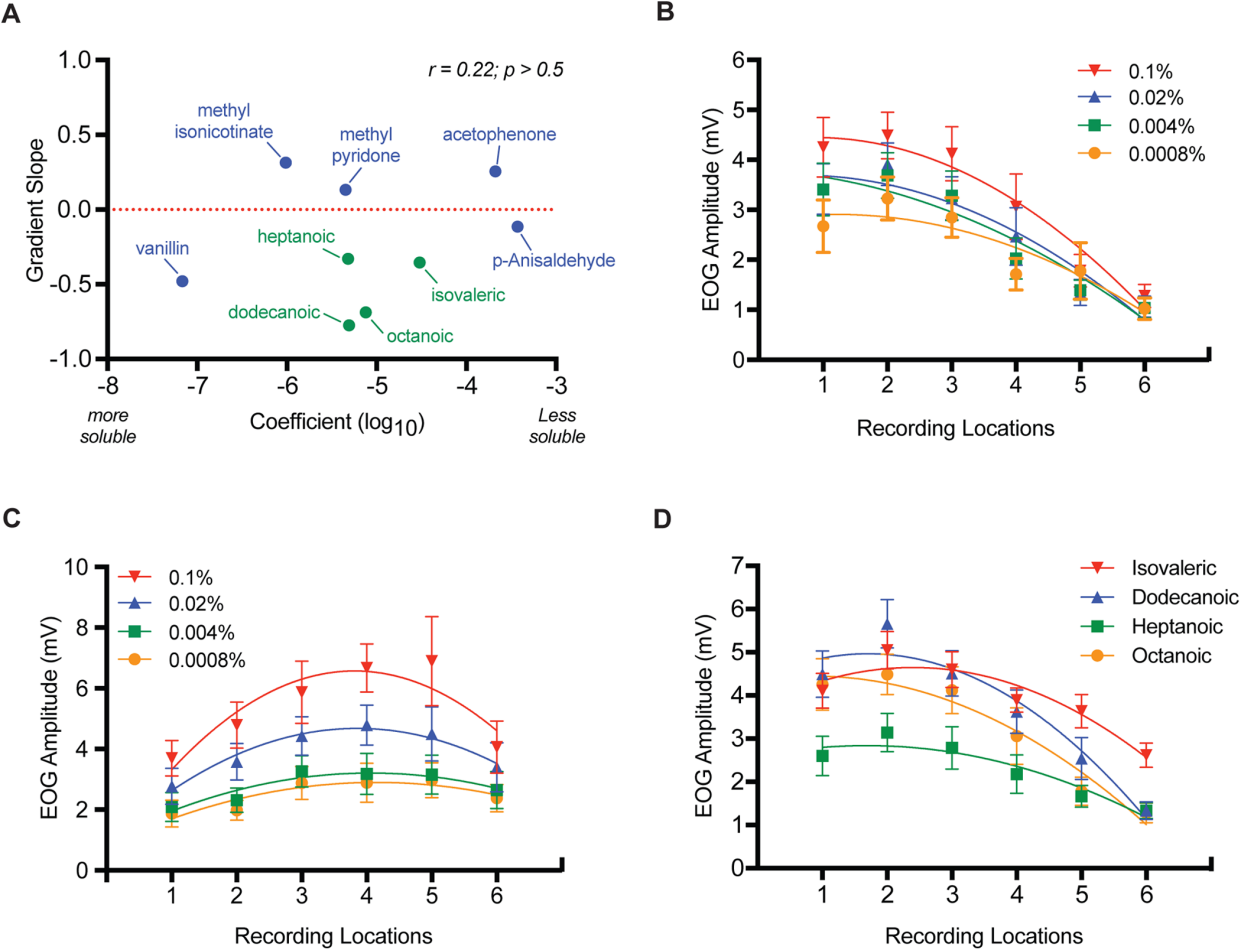
**The effects of mucus solubility, functional group and concentration on EOG gradients.** (A) For the highest odor concentrations only (see Results and below for justification), the relationship between the slope of the mean EOG response-gradient across six recording locations and air-mucus partition coefficients (Log_10_) is plotted. Pearson-r correlation and *P*-values are shown. (B) Mean (±s.e.m.; *n*=9 mice) EOG response amplitudes across six recording locations using half-log dilution series of octanoic acid. (C) Mean (±s.e.m.; *n*=12 mice) EOG response amplitudes across six recording locations using half-log dilution series of acetophenone. Note for B and C that concentration has little effect on the slope or shape of the response gradient across endoturbinate II_d_. (D) Mean (±s.e.m.; *n*=9–18 mice depending on odor) response gradients across six recording locations for four fatty acids (see text for explanation of stimulus concentrations and slope statistics).

To further examine the relationship between the pattern of odor responses across recording locations and odor solubility, the slopes of the linear fits to the EOG data for the nine odorants in Table 1 were compared to the log_10_ of their air-mucus partition coefficients (Fig. 3A). Only EOG data for the highest odor concentrations were included in the analysis, as justified below. Note the lack of a significant correlation between the gradient slope of an odor's EOG responses across recording locations and its mucus solubility. Thus, the most mucus-soluble odors were just as likely to have rising responses (positive linear slopes) moving from caudal to rostral recording locations as a falling response (negative linear slopes). A rising response means an odor caused a greater response outside the dorsal–central zone, while a falling response means an odor caused a greater response within the dorsal–central zone.

### Odor concentration and functional groups

As expected, EOG responses scaled with odor concentration. Importantly, concentration made little difference in the shape of the odor response gradient across recording locations, save for a flattening near threshold (Fig. 3C). For example, acetophenone displayed its characteristic pattern for all four of the concentrations used, which consisted of a rising EOG response moving from caudal to rostral locations that declined at the most rostral recording location (Fig. 3C). This resulted in the overall linear slopes of the response gradients for the different concentrations not deviating statistically from zero (not shown) or from each other (Friedman's statistic=0.733, *n*=9, *P*>0.8). Octanoic acid also displayed its characteristic declining response gradient as recordings moved from caudal to rostral location for all four concentrations tested. The median gradient slopes for octanoic acid were all significantly negative ranging from −0.32 (0.0008%, W=−45, *n*=9, *P*>0.004) for the lowest concentration to −0.65 (0.1%, W=−45, *n*=9, *P*>0.004) for the highest concentration and in this case were significantly different from each other (Friedman's statistic=21.13, *n*=9, *P*>0.0001; Fig. 3 B). That our chosen concentrations covered the entire lower range of EOG responses is borne out by the fact that still lower concentrations failed to give responses that were different from the response to blanks (data not shown). In our experience, even the purest blank elicits an EOG response presumably because of the well-documented mechanical sensitivity of olfactory sensory neurons (Grosmaitre et al., 2007).

Comparing response gradients at different stimulus concentrations for the other odors in Table 1 resulted in similar results (note vanillin, dodecanoic acid, isovaleric acid and methyl pyridone were tested at only one concentration). Thus, consistent with the untested assumptions of our previous studies, odor concentration does not appreciably alter response gradient shape (slope) across endoturbinate II_d_ (Coppola et al., 2014, 2017).

While odor solubility did not predict the shape of response gradients across recording locations on endoturbinate II_d_, the presence of a carboxyl function group apparently did. Four carboxylic acids were tested with carbon chains ranging from five (isovaleric acid) to 12 (dodecanoic acid) members (Fig. 3D). In each case, EOG responses declined as recording locations were moved from caudal to rostral (isovaleric acid median slope=−0.4, W=−137, *n*=17, *P*<0.0004; dodecanoic acid median slope=−0.66, W=−45, *n*=9, *P*<0.004; heptanoic acid slope=−0.29, W=−60, *n*=11, *P*<0.005; octanoic acid median slope=−0.65, W=−45, d.f.=9, *P*<0.004). The slopes of the gradients differed statistically though they all had similar shapes (Kruskal–Wallis statistics=17.0, *n*=46, *P*<0.0007). Vanillin was the only other odor with a response gradient like that of the fatty acids. As noted above, p-anisaldehyde had a negative response gradient slope but it was not significantly different from zero (W=−17, *n*=9, *P*>0.35).

## DISCUSSION

Despite the substantial body of work on the topic and its long history, the chromatographic theory of olfaction – including its very validity as a sensory coding mechanism – continues to be vigorously debated (cf. Rojas-Líbano and Kay, 2012; Cenier et al., 2013; Coppola et al., 2014, 2017; Courtiol et al., 2014, Scott et al., 2014). Reasons for our inability to gain consensus on the status of this construct include the facts that some of its elements have not been clearly articulated and its necessary and sufficient conditions have often been confounded; circumstances undoubtedly shared with many other persistent but dubious scientific postulates (Coppola et al., 2017). As pointed out previously, one essential condition for this proposed coding mechanism of olfaction to operate is for sorption patterns in the OE, which vary dramatically with odor solubility, to be correlated with the distribution of olfactory neuron odotypes. If this were not the case, i.e. if olfactory sensory neurons with a particular receptive-range were randomly distributed in the OE, a chromatographic distribution of an odor could not be detected, given the extreme convergence of these receptor projections onto the olfactory bulbs (Schoenfeld and Cleland, 2006; Scott et al., 2014; Coppola et al., 2017). Indeed, most olfactory receptor types converge on a pair of glomeruli, one medial and one lateral, in the olfactory bulb (Zou et al., 2009).

In apparent contradiction to this condition, our previous comparison of sorption patterns with the inherent response patterns of the OE showed no correlations within a turbinate, though a single recording location within the OE's dorsal–central region tended to respond more to highly mucus-soluble odors than either of two peripheral recording locations (Coppola et al., 2014, 2017). The current study extends these findings by: (1) labeling the dorsal–central zone (Zone 1) and comparing its boundaries to our standard electrophysiological recording locations, (2) focusing on an area of the OE with a marked sorption gradient, endoturbinate II_d_, and (3) using an extensive set of highly soluble odorants. In the last case, it was important to test odors at the extremes of mucus solubility since the chromatographic theory makes its clearest predictions about them and since this class of odors had been underrepresented in our previous studies (Coppola et al., 2014, 2017).

For this study, our CFD simulations were performed using airflow rates comparable to those during active sniffing as this condition is most relevant to normal odor sampling. Of the nine odors used for electrophysiological recording, three were chosen for CFD simulations on the grounds that they displayed diverse EOG response gradients on endoturbinate II_d_. The first odor, heptanoic acid, showed a steep decline in EOG responses moving from caudal toward rostral recording locations. The second, methyl isonicotinate, showed a steep rise (i.e. the opposite pattern) and the third, p-anisaldehyde had a flat response profile (non-significant linear slope). By contrast, the sorption gradients from our CFD simulations did not show any consistent relationship to these empirically derived EOG response gradients. Indeed, an important conclusion from our CFD simulations is that mucus-soluble odorants above the solubility of p-anisaldehyde, the least soluble chemical in our stimulus set, have very similar sorption gradients, dropping precipitously from caudal to rostral locations on endoturbinate II_d_. Thus, the sorption patterns from our CFD simulations should be applicable to any highly soluble odorant (see Scott et al., 2014).

To further test the conclusion that EOG response profiles in the OE are uncorrelated with particular stimulus' mucus solubility, we compared the air–mucus partition coefficients of each of our nine odorants to their EOG gradient slopes. Indeed, seven of our nine odorants had response gradient slopes across recording locations that were significantly different from zero. However, consistent with our CFD and EOG comparisons, there was not even a suggestion of a relationship between these variables.

Another limitation of our previous work on CT was the predominant use of a single odor concentration, which was a fairly strong stimulus judging by both our subjective evaluation (i.e. odor sampling) and the magnitude of the EOGs they evoked – near the upper quartile of the range for isoamyl acetate, for example (Coppola et al., 2017). Recent evidence suggests that a single high-sensitivity receptor type sets the threshold for odors and conversely generalist receptors that respond to high odor concentrations play less of a role perceptually (Dewan et al., 2018). Thus, our earlier studies might have missed correlations between CFD sorption gradients and responses from the most sensitive populations of olfactory sensory neuron. The four half-log unit concentrations used in the current study were chosen to include the majority of the response range for each odor (preliminary data not shown; see Materials and Methods). Results in each case suggest that while responses at any given recording location scale with odor concentration, the basic shape of the gradient changes very little across the entire receptive range of olfactory sensory neurons. These results validate the use of a single concentration in our previous studies and suggest that we are not missing relationships between CFD sorption gradients and EOG response gradients, which are only detectable at low odor concentrations. However, since the EOG technique integrates over thousands of olfactory sensory neurons, these conclusions are not definitive (Coppola et al., 2017).

To our knowledge, all previous EOG-based investigations of response maps within the OE were performed without the direct knowledge of olfactory receptor zone boundaries (Scott et al., 1996, 2000, 2014; Scott and Brierley, 1999; Scott, 2006; Coppola et al., 2014, 2017). This deficit in our knowledge was the motivation for immunolabeling the OE for NQO1 since the distribution of this enzyme is coextensive with the OE's dorsal–central zone (Gussing and Bohm, 2004**)**. Comparing the distribution of NQO1 immunolabeling with our recording sites revealed that our four caudal-most recording locations were in the dorsal–central zone, which previous evidence suggests is specialized for detecting olfactory stimuli involved in innate behavior (Kobayakawa et al., 2007) and is nearly the exclusive site of class I receptors (Niimura and Nei, 2007). Consistent with previous imaging, electrophysiological and heterologous expression assays, we found that the dorsal–central zone (locations one to four) was more responsive to fatty acids than areas outside this zone (Mori et al., 2006; Bozza et al., 2009; Saito et al., 2009). This conclusion was supported by the consistent negative slope of response gradients moving from recording locations one to six for each of the four fatty acids in our stimulus set. While the slopes of the gradients were significantly different, there was no obvious relationship between them and carbon chain length or solubility. Likewise, vanillin, a phenolic aldehyde, had a negative gradient slope like the fatty acids moving from location one to six (Mori et al., 2006). The significance of the focus of sensitivity for fatty acids and related compounds in the dorsal zone of the OE remains unknown. However, we have speculated previously that the OE response map may be represent an evolutionary contingent state without functional significance (Coppola et al., 2017).

The current study, together with our previous work (Coppola et al., 2017), provide scant support for the now classic theory that the rodent nose works like a chromatograph to promote odor coding at the level of the OE. Additionally, our data contradict one of the necessary conditions for the process to work: a correlation between odorant sorption patterns and olfactory sensory neuron odotype distribution. We have now tested several dozen odorants, spanning nearly the entire mucus solubility range, including a great variety of chemical types and odor qualia and can find no such relationship (Coppola et al., 2014, 2017). The current work extends these conclusions to odors with very high mucus solubility and may be applicable to all mucus-soluble odorants given this group's similarity in odor sorption patterns. The current study also extends our conclusions across the odor concentration range that can be measured by an ensemble recording method like the EOG.

Thus, we conclude, as previously, that a mode of olfactory coding based on a chromatographic effect appears to be implausible in the mouse (Coppola et al., 2014, 2017). Of course, we cannot prove the negative, a logical precept assuring that many ingenious but possibly erroneous ideas, like the chromatographic theory of olfaction, ‘never die, they just fade away’.

## MATERIALS AND METHODS

### Animals

Animal care and experimental procedures on female CD-1 strain mice (Charles River Labs Wilmington, MA, USA) followed the *Guide to the Care and Use of Laboratory Animals* (National Institutes of Health, USA) and were approved by the Randolph-Macon College Institutional Animal Care and Use Committees.

### NQO1 immunolabeling

Mice were deeply anesthetized with Nembutal, perfused with 0.1 M PBS (pH 7.2), and fixed by perfusion followed by immersion in fresh 4% paraformaldehyde. To create labeled whole mounts, heads from six mice were removed, hemisected along the midsagittal plane and placed in a steam bath of 10 mM citrate buffer for 1 h to retrieve antigen. Subsequently, hemi-heads were placed in blocking solution consisting of 7.5% rabbit serum in 0.02% TritonX-PBS (0.1 M) for 1 h followed by 2000-fold dilution of rabbit anti-NQO1 (#80588, Abcam USA) for 48 h and then washed. Specific labeling was visualized using an ABC kit for detecting rabbit primary antibody (Vector Labs, Burlingame, CA, USA) and DAB kit (Vector Labs) following the vendor's instructions.

For cryostat sections, heads were removed from five mice, decalcified for 4 h in RDO (Apex, Plainfield, IL, USA) and then cryoprotected by emersion in 40% sucrose overnight. Heads were frozen in dry-ice-cooled isopentane and cut in the coronal plane on a cryostat. Sections were mounted on subbed microscope slides placed in a steam bath of 10 mM citrate buffer for 1 h to retrieve antigen and reacted for 24 h in rabbit anti-NQO1 (Abcam) diluted 5000-fold (per above) and then washed. Specific labeling was visualized using an ABC kit for rabbit primary antibody (Vector Labs) and DAB kit (Vector Labs) following the vendor's instructions. As a control procedure for non-specific labeling, PBS was substituted for primary antibody in some assays. No additional control procedures were used since we were simply attempting to replicate the specific labeling shown by others for this antibody (Gussing and Bohm, 2004).

### EOGs

Our methods have been described in detail previously (Waggener and Coppola, 2007; Coppola et al., 2013, 2017; Barber and Coppola, 2015) and will only be briefly described here.

### Surgical preparation and electrophysiological recording

Immediately prior to electrophysiological recording, mice were killed with a lethal dose of Euthasol (70 mg per kg i.p.), which does not alter EOG responses (Scott and Scott-Johnson, 2002) and decapitated; skulls were then bisected along the midsagittal plane. Both hemi-heads were used for recording responses to odors after the nasal septum and overlying mucosa were resected to reveal the medial aspect of the endoturbinates. Only the dorsal branch of endoturbinate II_d_ was targeted in this study (Fig. 1).

Recordings took place within a Faraday cage covered with plastic sheeting. This chamber was suffused with humidified air from two commercial forced-air units such that humidity was maintained at >98% around the preparation. The positive pressure created by the humidifiers also served to exhaust the chamber of spent odors.

EOGs were recorded at six equally spaced intervals (∼1 mm) along the medial face of endoturbinate II_d_ near its dorsal edge to reveal any intrinsic spatial patterns of response (Fig. 1). Interval length varied slightly due to differences in animal size. Odors were only tested once at each location at a particular concentration and a different set of subjects (*n*≥9) was used for each odor. For each subject, all locations were targeted for recording with a counterbalanced ordering of locations among animals. For the odors used at multiple concentrations, the sequence at a particular recording site was always lowest to highest concentration. Interstimulus interval was held to a minimum of 50 s and the entire recording period was kept to within 45 min of death.

A recording electrode was positioned on the medial surface of endoturbinate II_d_ using a three-axis manipulator. An indifferent electrode was placed on the frontal bone at its intersection with the cribriform plate and immobilized with a magnetic clamp. Recording electrodes consisted of Ag/AgCl wires inside glass capillaries that had been pulled to approximately 50 µm tip diameter and filled with 0.05% agar in 0.1 M PBS. The indifferent electrodes consisted of Ag/AgCl wire inside a 500 µl pipette tip filled as above. Electrodes were connected to the inputs of an Iso-DAM8A DC Amplifier (World Precision Instruments, Sarasota, FL, USA). The output of the amplifier was sampled at 20 Hz by a PowerLab/8SP physiograph (AD Instruments, Colorado Springs, CO, USA) which provided A/D conversion, display, and recording. EOG maximum amplitude was the dependent variable for all experiments and was measured by manual cursor placement at the nadir of the EOG trace using LabChart software (v7.2.5).

### Stimulation protocol

Odors were delivered to the mucosal surface via a 0.5 s pulse of air (700 ml/min) from the headspace above a 10 ml mixture of odorant dissolved in mineral oil contained in a 25 ml vial except in the case of vanillin and dodecanoic acid. For these latter odorants, which are solid at room temperature, 100 mg of solid was placed in a vial without mineral oil. The carrier gas was charcoal-filtered room-air humidified prior to entering the stimulus apparatus. A custom unit consisting of a computer, software, interface and olfactometer (Knosys Inc., Lutz, FL, USA) controlled stimulus duration and timing. However, odor type and concentration were selected by manually switching the reservoir vial that was in line with the odor port. The interstimulus interval was held to a minimum of 50 s for all experiments.

The odor delivery port consisted of a 3 cm long, 3.5 mm diameter glass tube connected by a 3 cm long Teflon tube to the odor reservoir vial. A three-axis micromanipulator was used to position the odor port. A rigid guide-hair affixed to the end of the odor port allowed us to maintain a consistent standoff distance of 10 mm and an angle of 45° in relation to the surface of the OE.

### Choice of the stimulus set

The nine stimuli used in this study were all single molecules at or near the highest purities (>97% to >99%), commercially available from Sigma-Aldrich. Six of the odors (vanillin, methyl isonicotinate, octanoic acid, methyl pyridine, heptanoic acid and p-anisaldehyde) were selected from a set of 66 recently used in a study of CT in the rat (Scott et al., 2014). These six represent a sampling from eight of the most mucus-soluble odors in this previous study (with log air-mucus partition coefficients below −3.4). While these are extremely water- (and mucus-) soluble odorants, water solubility alone does not completely capture the parameter of ‘sorptiveness’ as regards interaction of an odorant with the mucus layer that lines the OE (Kurtz et al., 2004). Thus, the set of six odors were selected based on their high air/mucus partition coefficients which were calculated from their air/water and octanol/water partition coefficients obtained from the U.S. EPA EPI Suite as described by Rygg et al. (2017) (Table 1). Two additional odors were used to expand our sample of fatty acids: isovaleric acid, a mucus-soluble short-chain species and dodecanoic acid, a markedly less mucus-soluble, long-chain species (Table 1). Finally, acetophenone was included in our odor set for comparison with our previous results (Coppola et al., 2014).

The stated stimulus concentrations are v/v dilutions of odor in mineral oil, except for vanillin and dodecanoic acid, which were used in undiluted solid form, as noted above, since they are not miscible in mineral oil. All odors were tested at 0.1% concentration and all but vanillin, dodecanoic acid, methyl pryridone and isovaleric acid were tested at other concentrations between 0.1% and 0.0008% to determine if response patterns across recording location changed with concentration. To systematically illustrate the general result, octanoic acid and acetophenone were tested at four concentrations: 0.1%, 0.02%, 0.004% and 0.0008%. These concentrations were chosen because preliminary studies (data not shown) established that they encompass nearly the entire response range for these odorants. The exact odor concentrations delivered to the OE surface were undetermined as they would be difficult to measure and are not critical to the hypothesis under test.

### Computational fluid dynamics simulations

CFD simulations of airflow and odorant deposition in the right nasal cavity of a 38.8 g CD-1 strain female mouse (Charles River Laboratories) were conducted using our previously developed model (Coppola et al., 2014, 2017). Comparisons of this individual to our archive of histological sections and morphometric analyses of multiple specimens from one of our previous studies (Coppola et al., 2014) allow us to conclude that it is generally representative of the CD-1 mouse strain. Three odorants, heptanoic acid, methyl isonicotinate and p-anisaldehyde, which showed diverse patterns of EOG responses, were selected for use in the simulations.

Briefly, the anatomical nasal airway model was reconstructed from 25 μm resolution MRI scans using previously described methods (Craven et al., 2007; Ranslow et al., 2014). An unstructured hexahedral CFD mesh was generated using the *snappyHexMesh* utility available in OpenFOAM (version 2.4). The CFD mesh contained approximately 18 million computational cells, which was previously determined to be adequate based on the results of a CFD mesh refinement study (Coppola et al., 2017). Boundary conditions were specified as in previous nasal airflow studies (Craven et al., 2009, 2010; Pang et al., 2016; Coppola et al., 2017) and a steady-state CFD simulation of inspiratory airflow during a quasi-steady sniff at 100 ml/min (Challis et al., 2015) was conducted using the SIMPLE (Semi-Implicit Method for Pressure-Linked Equations) solver available in OpenFOAM. As justified by Rygg et al. (2017), the present CFD simulations assume that the walls of the nasal cavity are rigid and that the thin mucus lining on the nasal airways has a negligible effect on the intranasal airflow. Airflow is modeled as laminar, which is well-justified based on the fact that the magnitude of the non-dimensional Reynolds number (approximately 50–250) is well below the criterion of 2000 when internal flows typically transition from laminar to turbulent (White, 2011). Finally, we assume that the airflow during sniffing is quasi-steady because the magnitude of the non-dimensional Womersley number is in the range of 0.2–0.6, which is well below the criterion of unity that indicates when unsteady flow phenomena become significant (see Rygg et al., 2017 for further discussion and justification of this quasi-steady assumption).

As in previous work (Lawson et al., 2012; Rygg et al., 2017; Coppola et al., 2017), quasi-steady simulations of nasal odorant deposition were performed for three odors (heptanoic acid, methyl isonicotinate and p-anisaldyde) with an inlet concentration of 1 μmol/m^3^. The air-mucus odorant partition coefficients (Table 1) were calculated as in Rygg et al. (2017). Importantly, calculation of the partition coefficients utilized the correlation of Scott et al. (2014) that was developed based on the experimental odor mucus solubility data of Kurtz et al. (2004).

ParaView (version 5.1) was used to analyze and post-process the raw CFD simulation results. Steady-state flow streamlines were computed to visualize the inspiratory nasal airflow patterns and contours of odorant flux (sorption) were visualized on the walls of the airway. Quantitative odorant flux values were extracted along endoturbinate II_d_ at approximately the same spatial locations as the EOG recordings (Fig. 1A).

### Statistics

All statistics were performed in Microsoft Excel and Prism 8 for Mac OS (GraphPad Software, San Diego, CA, USA). Since responses across recording locations were repeated measures, linear slopes were calculated separately for each animal in Microsoft Excel and exported to Prism. Departures of the average slopes from zero were tested by the method of Wilcoxin. Comparisons of average slopes between concentrations or odors were performed by Friedman's test for paired comparisons and Kruskal–Wallis' test for independent samples. There were no *a priori* hypotheses concerning post-hoc comparisons, so they are not reported. The targeted minimum sample size of nine mice per odorant was based on EOG variability measures from Coppola et al. (2017).
